# Peripartum Management of Congenital Fiber Type Disproportion Myopathy With Severe Restrictive Lung Disease

**DOI:** 10.7759/cureus.32019

**Published:** 2022-11-29

**Authors:** Shibinath Velutha Mannil, Shamantha Reddy, Erik B Romanelli

**Affiliations:** 1 Obstetric Anesthesiology, Albert Einstein College of Medicine, Bronx, USA; 2 Addiction Medicine, Baptist Memorial Hospital, Memphis, USA

**Keywords:** malignant hyperthermia (mh), labor and birth, peri partum, restrictive lung disease, pregnancy, myopathy

## Abstract

Congenital myopathies raise unique challenges for anesthesiologists during labor and delivery. Apart from having a risk for malignant hyperthermia, this patient population can present with severe restrictive lung disease in the third trimester. Scoliosis and weak pelvic muscles could make regional anesthesia difficult. The common complications in pregnancy include premature labor, preterm delivery, spontaneous abortion, a prolonged first stage of labor, and uterine atony. We report a case of 28-year-old primigravida of 37 weeks gestation diagnosed with congenital fiber type disproportion successfully managed by a team of high-risk obstetricians, anesthesiologists, and pulmonologists. The patient was closely monitored with serial arterial blood gas to determine carbon dioxide retention in a high-risk labor floor with a backup operating room for cesarean delivery. We reserved a malignant hyperthermia cart and a postpartum hemorrhage cart for emergencies. Our patient was able to deliver vaginally with the help of ultrasound-guided regional anesthesia despite having severe restrictive lung disease and scoliosis. We emphasize a multi-disciplinary team approach for a successful outcome for this patient population.

## Introduction

This article was previously presented as a meeting abstract at the 2022 Society of Obstetric Anesthesiology and Perinatology (SOAP) in Chicago, USA, on May 11-15, 2022.

Congenital myopathies (CM) encompass a spectrum of inherited genetic disorders, which affect muscle tone and contraction. In adults, the clinical course is often static or slowly progressive. In this case report, we describe the peripartum management of a patient with a known diagnosis of a particular congenital myopathy known as congenital fiber-type disproportion (CFTD). CFTD can have multiple inheritance patterns with defects mapped to SEPN1, ACTA1, and TPM3 genes [1,2]. Clinical symptoms in the mild form include facial dysmorphisms and skeletal muscle weakness around the shoulder, hip, upper arms, and thighs [3,4]. If severe, a patient may suffer from ophthalmoplegia, progressive scoliosis, dysphagia, dilated cardiomyopathy, and respiratory muscle weakness [3,4]. However, literature on the anesthetic management of CMs in pregnancy is limited. By describing (I) the goals of pre-anesthesia evaluation, (II) the potential need for malignant hyperthermia (MH) precautions [5], and (III) the use of a multi-disciplinary team to manage CMs in pregnancy, we intend to fill the knowledge gap for effective management of CMs in pregnancy [6].

## Case presentation

**Figure 1:**
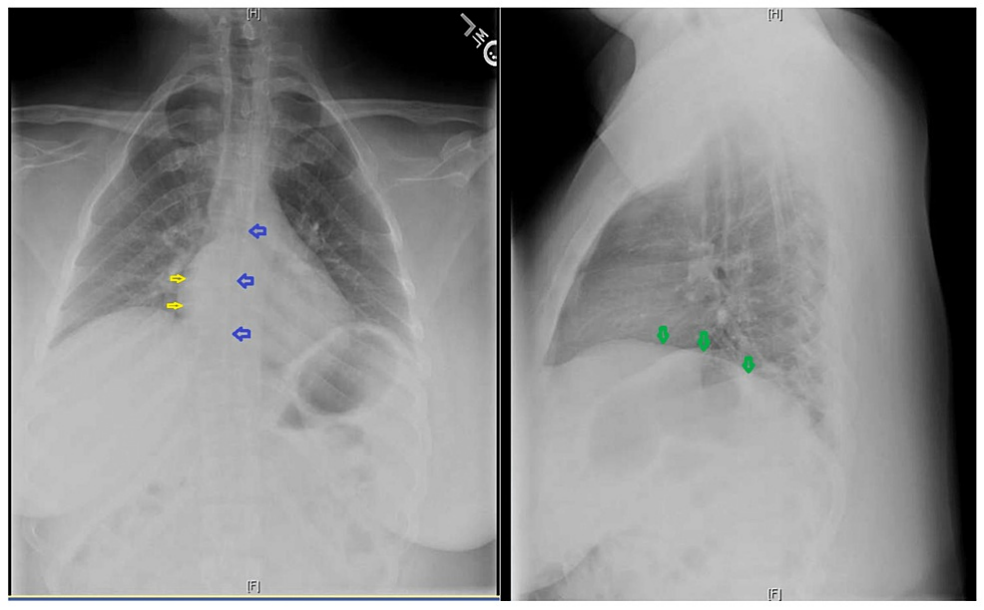
Chest X-ray AP and lateral views Chest X-ray anteroposterior (AP) and lateral views (L) AP view demonstrating a bell-shaped thoracic cage. Blue arrows represent scoliosis. Yellow arrows represent the areas of mild atelectasis. In the lateral view, green arrows demonstrate mild flattening of the diaphragm from congenital fiber-type disproportion myopathy.

**Figure 2:**
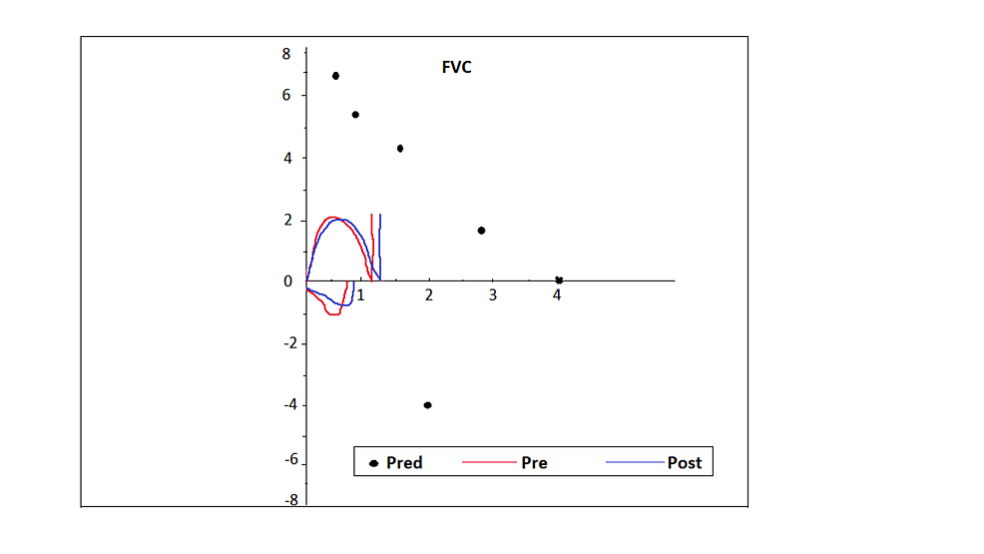
Flow-volume curve Demonstrating severe restrictive pattern. The black dots represent the predicted flow-volume curve, and the red and green lines represent the pre and post-bronchodilator therapy flow-volume curve, respectively. There was no significant post-bronchodilator response.

A 28-year-old primigravida with a history of CFTD was admitted for labor induction at 37 weeks for preeclampsia with severe features. Past medical history was significant for obstructive sleep apnea (OSA), requiring intermittent continuous positive airway pressure (CPAP) with pressure support of 5-8 cmH_2_O, mild asthma, and chronic hypertension (HTN). Past surgical history was significant for sleeve gastrectomy for a prior BMI of 58.8 (current BMI was 36). She reported episodic generalized weakness and difficulty walking downstairs and combing her hair. Heart rate was 88/min, blood pressure 160/92 mmHg, respiratory rate 27/min, and blood oxygen saturation (SpO2) 93% on room air. Airway exam revealed Mallampati class-III, adequate mouth opening, high-arched palate, and thyromental distance (TMD) of less than three finger breadths. Weakness of bilateral sternocleidomastoid, iliopsoas, and limitation in upward gaze was previously documented by neurology. Chest X-ray (CXR) reported atelectasis and mild thoracic scoliosis (Figure 1). A pulmonary function test (PFT) demonstrated severe restrictive lung disease (forced vital capacity - 1.11 L) with reduced diffusion capacity. Venous blood gas (VBG) reported a baseline partial pressure of carbon dioxide (PCO_2_) of 72.8 mmHg. An echocardiogram revealed an ejection fraction (EF) of 65% with no regional wall abnormalities. Levetiracetam was used instead of magnesium sulfate for seizure prophylaxis because of concerns for respiratory depression. Bilevel positive airway pressure (BiPAP) was initiated with pressure support of 6 cm H2O and was titrated according to PCO_2_ value and the patient's respiratory effort. Figure 2 shows the flow-volume curve. Arterial blood gases (ABG) were obtained every two hours. Hypertensive episodes were initially managed with IV labetalol. Ultrasound-guided dural puncture epidural (DPE) was placed in a sitting position at the L4-L5 level. The procedure was challenging due to her scoliosis, obesity, and bilateral iliopsoas weakness. Patient-controlled epidural analgesia was administered using 0.0625% bupivacaine and fentanyl 2 mcg/mL at 12 cc/hr. She delivered vaginally and received oxytocin, tranexamic acid (TXA), and misoprostol immediately postpartum. The patient was subsequently weaned off from BiPAP on postpartum day one.

## Discussion

Congenital fiber-type disproportion occurs with an incidence of 1 in 50000 individuals. The condition is diagnosed with type-1 muscle fiber hypotrophy of at least 12% and clinical features of myopathy [7]. Approximately 25% of patients experience severe symptoms such as respiratory failure, ophthalmoplegia, dysphagia, dilated cardiomyopathy, and kyphoscoliosis [8]. Congenital myopathies have historically been associated with motor and pulmonary function exacerbation during pregnancy [9]. Specifically, early-onset and progressive myopathies (like CFTD) have been associated with greater morbidity in pregnancy [10]. As was observed in our CFTD patient, patients with congenital myopathies associated with diaphragmatic and respiratory muscle involvement are at greater risk for needing ventilatory support. Myopathic patients' worsening of muscle weakness, especially in the third trimester, is attributed to the effect of rising progesterone on cellular potassium homeostasis [11,12]​​​​​.

The goal of the pre-anesthetic evaluation should be accurately assessing disease severity and optimizing comorbidities with the assistance of a multi-disciplinary team. Neuraxial analgesia/anesthesia (barring contraindication) with a CSF-confirmatory technique (i.e., CSE or DPE) is deemed preferable in obstetric settings [13]. There are no contraindications for regional anesthesia in stable CMs [6]. In a CM patient with diaphragmatic involvement, (early) neuraxial placement should be strongly advocated given the increased risks associated with general anesthesia (i.e., malignant hyperthermia susceptibility, difficult ventilatory management for severe restrictive lung disease, prolonged postoperative mechanical ventilation). Multidisciplinary consultation will help guide the need for additional workup, which can include (but is not limited to): 1) Chest X-ray (to rule out pulmonary infiltrates, atelectasis, cardiac enlargement, and abnormal spine curvature), 2) 12-lead EKG (for assessment of cardiac muscle involvement and ruling out arrhythmias related to heart block) and/or transthoracic echocardiogram (for ruling out pre-existing cardiomyopathy), and 3) spirometry (for assessment of restrictive lung disease severity. The current recommendation is to perform a comprehensive PFT annually and polysomnography every two years in patients with CMs [8,14]​​​.

Once the multidisciplinary team has determined the ideal mode of delivery, an anesthesia plan can be formulated for either vaginal delivery or cesarean delivery. Anesthetic plans in an urgent or emergent cesarean delivery setting should also be reviewed with team members, emphasizing the preferability of neuraxial anesthesia (unless the patient develops acute respiratory insufficiency requiring ventilator support). Anesthetic medications should be chosen with an awareness of the potential for MH in patients with RYR1 mutations, and an MH cart should be available in the OR [6]. We recommend placing early DPE or combined spinal and epidural (CSE), as these techniques have lower failure rates compared to a straight epidural [15]. Also, it is prudent to keep a low threshold to replace epidurals failing to achieve adequate analgesia, given the higher likelihood of failure to convert for surgical anesthesia and associated general anesthesia risks in CFTD. For most CMs, including CFTD, succinylcholine and volatile anesthetics have not been documented for fear of MH provocation. Therefore, it seems prudent not to use these agents for CFTD patients. They are also at higher risk of joint dislocation (particularly hip), so positioning efforts should be given additional attention [16]. In the labor and delivery room, measures for managing sudden respiratory compromise, including a difficult airway cart, should be readily available. It is essential to have a postpartum hemorrhage cart available for the labor and delivery floor and operating rooms since this population is theoretically at greater risk for lower segment uterine atony due to myopathy.

According to the American Thoracic Society, forced vital capacity <35% of predicted is considered very severe and can develop cor-pulmonale. Most patients require non-invasive ventilation (NIV) to compensate for respiratory muscle weakness. Our patient had worsening dyspnea, especially in the third trimester, and required higher pressure support than her usual CPAP requirements before pregnancy. Women with restrictive lung disease can tolerate pregnancy reasonably well, but many have premature deliveries [17]. Adequate fetal growth can be achieved by optimal antenatal care [18]. During the intrapartum period, early administration of NIV and serial monitoring of oxygenation are recommended [19]. Patients with CM should be monitored throughout the pregnancy by a high-risk maternal and fetal medicine team for complications like premature labor and preterm delivery, spontaneous abortion, a prolonged first stage of labor, uterine atony, polyhydramnios, abnormal presentation, and increased incidence of neonatal death [12,19].

## Conclusions

In the peripartum period, we emphasize the importance of a multidisciplinary team approach, especially for decisions regarding the mode of delivery and comorbidity optimization. Anesthetic plans for labor analgesia and/or surgical anesthesia can be subsequently tailored to the individual patient. FVC values above one liter can facilitate vaginal delivery even though there are no clear guidelines for a cut-off value. In general, for the majority of patients, the outcome is favorable and the disease trajectory is relatively stable.
